# Primary Care–Based Digital Health–Enabled Stroke Management Intervention: Long-Term Follow-Up of a Cluster Randomized Clinical Trial

**DOI:** 10.1001/jamanetworkopen.2024.49561

**Published:** 2024-12-13

**Authors:** Jie Tan, Enying Gong, John A. Gallis, Shifeng Sun, Xingxing Chen, Elizabeth L. Turner, Siran Luo, Jingying Duan, Zixiao Li, Yilong Wang, Bolu Yang, Shiyu Lu, Shenglan Tang, Janet P. Bettger, Brian Oldenburg, J. Jaime Miranda, Biraj Karmacharya, Sanjay Kinra, Ruitai Shao, Shah Ebrahim, Lijing L. Yan

**Affiliations:** 1 School of Public Health, Wuhan University, Wuhan, China; 2 Global Health Research Center, Duke Kunshan University, Kunshan, China; 3 School of Population Medicine and Public Health, Chinese Academy of Medical Sciences and Peking Union Medical College, Beijing, China; 4 State Key Laboratory of Respiratory Health and Multimorbidity, Chinese Academy of Medical Sciences and Peking Union Medical College, Beijing, China; 5 Duke Global Health Institute, Duke University, Durham, North Carolina; 6 Department of Biostatistics and Bioinformatics, Duke University, Durham, North Carolina; 7 Department of Neurology, Beijing Tiantan Hospital, Beijing, China; 8 Baker Heart and Diabetes Institute, Melbourne, Victoria, Australia; 9 School of Psychology and Public Health, La Trobe University, Melbourne, Victoria, Australia; 10 CRONICAS Centre of Excellence in Chronic Diseases, Universidad Peruana Cayetano Heredia, Lima, Peru; 11 Sydney School of Public Health, Faculty of Medicine and Health, University of Sydney, Camperdown, New South Wales, Australia; 12 Dhulikhel Hospital–Kathmandu University Hospital, Kathmandu University School of Medical Sciences, Dhulikhel, Nepal; 13 London School of Hygiene & Tropical Medicine, London, United Kingdom

## Abstract

**Importance:**

Despite evidence of the short-term benefits of multicomponent primary care–based interventions, their long-term effects are unproven.

**Objective:**

To evaluate the long-term outcomes of a system-integrated technology-enabled model of care (SINEMA intervention) for stroke management for systolic blood pressure (BP) and other outcomes among patients with stroke in China.

**Design, Setting, and Participants:**

This long-term follow-up included community-dwelling clinically stable surviving participants with stroke in an open-label cluster randomized clinical trial. Of 218 villages from Nanhe County in Hebei, China, an area with suboptimal health care resources and stroke prevalence doubling the national average, 50 villages (clusters) were recruited between June 23 and July 29, 2017, and randomized in a 1:1 ratio to an intervention or a control arm (usual care). The intervention lasted 1 year (to July 31, 2018), with a posttrial observational follow-up conducted from October 1, 2022, to August 27, 2023.

**Interventions:**

Village doctors were provided with training, performance-based incentives, technical support, and customized mobile health tools to deliver monthly follow-up to patients. Patients also received daily voice messages emphasizing medication adherence and physical activity. No intervention was requested or supported during the posttrial period.

**Main Outcomes and Measures:**

Between-arm differences in intention-to-treat analyses of individual-level changes from baseline to long-term posttrial in systolic BP (primary outcome) and stroke recurrence, diastolic BP, BP control, antihypertensive medication use and regimen adherence, and disability (secondary outcomes).

**Results:**

Among a total of 1042 stroke survivors, 44 (4.2%) were lost to follow-up and 998 (mean [SD] age at baseline: 65.0 [8.2] years; 544 [54.4%] men) completed posttrial assessment at a mean (SD) period of 66.6 (3.7) months (5.5 years) after baseline. The multicomponent intervention was associated with an estimated between-arm net reduction in systolic BP of −2.8 (95% CI, −5.3 to −0.3) mm Hg (*P* = .03). Most secondary outcomes showed a tendency toward lasting effects, with a notable absolute net reduction of 6.0 (95% CI, −11.3 to −0.7) percentage points and risk ratio of 0.77 (95% CI, 0.61-0.99) for stroke recurrence. In subgroup analyses, significant between-arm differences were observed among women and people with lower educational attainment, lower income, and higher use of and adherence to medications.

**Conclusions and Relevance:**

In this long-term follow-up of a cluster randomized clinical trial, the 1-year intervention was associated with significantly reduced systolic BP and stroke recurrence at 5.5 years, providing evidence of long-term health and inequity-reducing benefits and holding promise for scaling up of the intervention in resource-limited settings.

**Trial Registration:**

ClinicalTrials.gov Identifier: NCT05792618

## Key Points

**Question:**

Can a 1-year primary care–based, mobile technology–enabled intervention among patients with stroke in rural China lead to long-term benefits in blood pressure and other outcomes?

**Findings:**

In this secondary analysis of a cluster randomized clinical trial including 998 patients in 50 villages, after 5.5 years, the intervention compared with usual care was associated with a significantly lower mean systolic blood pressure (−2.8 mm Hg between-arm difference) and a lower proportion of patients with recurrent strokes.

**Meaning:**

These results hold promise for scaling up and widespread adaptation in resource-limited settings.

## Teaser

This long-term follow-up of a cluster randomized clinical trial investigates whether the positive outcomes of a system-integrated technology-enabled model of care on systolic blood pressure and stroke recurrence persist among patients with stroke over an extended postintervention period.

## Introduction

High blood pressure (BP) is a critical and modifiable risk factor for the prevention and management of stroke.^1^ Lowering BP has been consistently demonstrated to reduce the risk of subsequent stroke events.^2,3^ Several cluster randomized clinical trials (cRCTs) in low- and middle-income countries^4,5,6,7,8,9,10^ have underscored the effectiveness of interventions based in primary care and community settings for enhancing BP control among individuals diagnosed with hypertension or cardiovascular diseases. However, only a few studies specifically targeted patients with stroke.^11,12,13^ In addition, these interventions often spanned only 1 to 2 years, leaving uncertain the long-term sustainability of their effects after the intervention was completed.

To address the large burden of stroke, the leading cause of death and disability in rural China,^14,15^ our group designed and implemented a system-integrated, technology-enabled model of care (SINEMA) to provide a primary care–based intervention for improving stroke management.^16^ Through a cRCT involving 50 clusters (villages) conducted from June 23, 2017, to August 17, 2018, among patients with stroke, significant reductions in the primary outcome of systolic BP were observed.^11,17^ The present study aims to investigate whether the positive effects on systolic BP and stroke recurrence observed at the end of the SINEMA intervention persist over an extended postintervention period.

## Methods

### Study Design, Setting, and Participants

This study is a 5.5-year postbaseline (4.5-year posttrial) follow-up of the primary care–based, open-label, 2-arm SINEMA cRCT conducted in Nanhe County, Hebei, China, where stroke prevalence rates doubled the national average.^14^ Nanhe County had a total population of about 350 000 individuals living in 218 villages in 8 townships. The detailed study design of the cRCT has been described and published previously,^16^ and the protocols for both the main trial and the follow-up assessment are in Supplement 1. Briefly, the original trial included 10 villages (clusters) from each of 5 of 8 townships, resulting in a total of 50 villages.^18^ People with a history of stroke diagnosed at county- or higher-level hospitals and in a clinically stable condition with at least basic communication abilities were eligible. Participants were excluded if they were bedridden, had severe life-threatening diseases with an expected life span shorter than 6 months, or did not plan to live in the villages at least 6 months in the next year. Recruitment took place between June 23 and July 29, 2017.

Randomization was performed after baseline data collection by an independent biostatistician (J.A.G.). Fifty villages were randomized, with stratification by township, in a 1:1 ratio to either the intervention arm or the control arm (usual care). The study was approved by the ethics boards of the Chinese Academy of Medical Sciences and Peking Union Medical College (and Duke University), with written informed consent obtained from all participants, including the signing of new forms for this secondary analysis. Reporting of the study adheres to the Consolidated Standards of Reporting Trials (CONSORT) guideline with extension for cRCTs.

### Intervention

Participants in the intervention arm were offered the SINEMA intervention, a primary care–based strategy for stroke management, incorporating elements for both health care professionals and patients. Village doctors (primary care practitioners in rural China with basic professional training and the right to prescribe medicines in essential medicine formularies) were provided with 1-day training, performance-based financial and nonfinancial incentives, and the SINEMA app to conduct monthly follow-up visits with patients. These visits included BP monitoring, stroke symptom evaluation, monitoring adherence to medication use, and physical activity encouragement. Concurrently, patients were afforded monthly follow-ups and daily voice messages for those with phone access, automatically dispatched at no cost to them, aimed at reinforcing medication regimen adherence and physical activity. Design of the intervention was guided by the chronic care model^16,19^ and logic model^20^ and informed by extensive preimplementation field research and community engagement, published in 3 reports including app development,^21^ phone messaging,^22^ and overall intervention and trial design.^16^ The active phase of the intervention was from June 23, 2017, to July 31, 2018. Although intervention support ended in July 2018, village doctors could voluntarily continue to use the SINEMA app for free and offer these services without incentives. In contrast, the control arm participants received usual care, necessitating proactive care seeking. This included, for patients diagnosed with hypertension and/or type 1 or 2 diabetes, access to quarterly follow-ups and generalized health education as part of a broader national public health initiative.^23^

### Assessments and Outcome Measures

Staff from the Center for Disease Control in a neighboring county conducted all data collections at baseline, 1 year, and the recent 5.5-year posttrial follow-up conducted from October 1, 2022, to August 27, 2023. They were blinded to cluster allocation status and not involved in the implementation of the intervention. They received training to adhere to the same standardized protocol in all villages and participants and across the 3 assessments. The assessments included face-to-face interviews and physical measurements, with data entry facilitated through an online platform (Qualtrics).

In October 2022, near the end of the 3-year COVID-19 pandemic, all surviving participants, regardless of their follow-up status at 1 year, were invited to participate in a posttrial follow-up assessment. After completing 34 of 50 clusters, we had to delay data collection for the remaining 16 clusters until May 2023 when the massive COVID-19 outbreak from the winter of 2022 to spring of 2023 subsided. An additional effort was made in August 2023 to follow up participants who were not reached previously (eFigure 1 in Supplement 2).

We remeasured the original outcomes 5.5 years after the baseline (4.5 years following the intervention’s cessation). Blood pressure was measured on the right upper arm while the participants were seated following 5 minutes of quiet rest, using a validated electronic BP monitor (HEM-7052; Omron Healthcare Inc).^11^ Two measurements were obtained and the mean value was calculated. If the difference between the first 2 systolic BP readings exceeded 10 mm Hg, a third measurement was taken, and the last 2 readings were used for calculation.

We examined 5 prespecified secondary outcomes,^24^ including changes in diastolic BP, self-reported antihypertensive medication use and adherence (measured as 0 on the 4-item Morisky Green Levine Scale, with lower scores indicating higher adherence; people who received a score of 0 were defined as having adherence to the regimen),^25^ disability (measured by the modified Rankin Scale, with scores ranging from 0 (no symptoms) to 5 (severe disability); people with a score of ≥3 were categorized into the "moderate to severe disability" group.),^26^ and nonfatal stroke recurrence (self-reported by participants indicating any nonfatal stroke events). We also conducted post hoc analyses on systolic BP control (defined as systolic BP <140 mm Hg) and BP control (defined as systolic BP <140 mm Hg and diastolic BP <90 mm Hg). Other metrics, such as cause-specific mortality, were assessed through data linkage methods and will be reported separately.

### Statistical Analysis

For analyses of posttrial outcomes, we followed the statistical analysis plan of the main trial, published elsewhere.^24^ The intention-to-treat principle was used for all analyses. For continuous variables, we applied mixed-effects linear regression models to estimate between-arm differences in mean outcomes at 1 and 5.5 years. Each model included a random intercept for village (cluster) and fixed effects for intervention arm, baseline systolic BP, township, sex, age, and month of interview. We removed outliers of systolic and diastolic BP in the main analyses based on an a priori decision to remove those that were more than 2 IQRs above the third quartile or below the first quartile,^24^ but sensitivity analysis was performed by including these outliners. When the mixed-effects model did not converge, we used the generalized estimating equations (GEE) method with a gaussian model and an identity link. For binary outcome variables, GEE was adopted for a population-averaged interpretation. We used a Poisson model with log link and robust SEs to obtain risk ratios (RRs) and with identity link to obtain risk differences.^27,28^ All GEE models used an independence working correlation.^29^ If GEE models did not converge, we used a marginal standardization approach by fitting a binomial model with a logit link to obtain risk differences and RRs.^28^

We conducted the prespecified subgroup analysis for the primary outcome of systolic BP by sex, age, educational attainment, and disease duration since the latest stroke event at baseline.^11^ To further understand the potential differences in long-term effectiveness, post hoc subgroup analyses were performed by other baseline characteristics, including annual household income, status of antihypertensive medication use and adherence, self-reported hypertension, stroke type, disease duration since the first stroke event, level of systolic BP, disability status, and experience of stroke recurrence since diagnosis. Interactions between treatment effect and subgroups were assessed using a test of the 2-way interaction effects.

Sensitivity analyses were performed involving additional adjustment for covariate posttrial follow-up duration, covariates that were statistically imbalanced (*P* < .05) between arms at baseline, covariates associated with significant differences (*P* < .05) between survivors at 5.5 years and those lost to follow-up or decedents, and including all outliers (which were excluded from the main analyses).^24^ We also conducted sensitivity analyses by restricting the sample to participants who completed all 3 assessments and by excluding 19 participants who were followed up in August 2023, considering the potential seasonal variations of BP. All analyses were conducted using Stata, version 17.0 (StataCorp LLC), and codes were independently validated by biostatisticians at Duke University (J.A.G. and S.S.). All hypothesis tests were 2 sided, and *P* < .05 was considered statistically significant.

## Results

**Figure 1.**
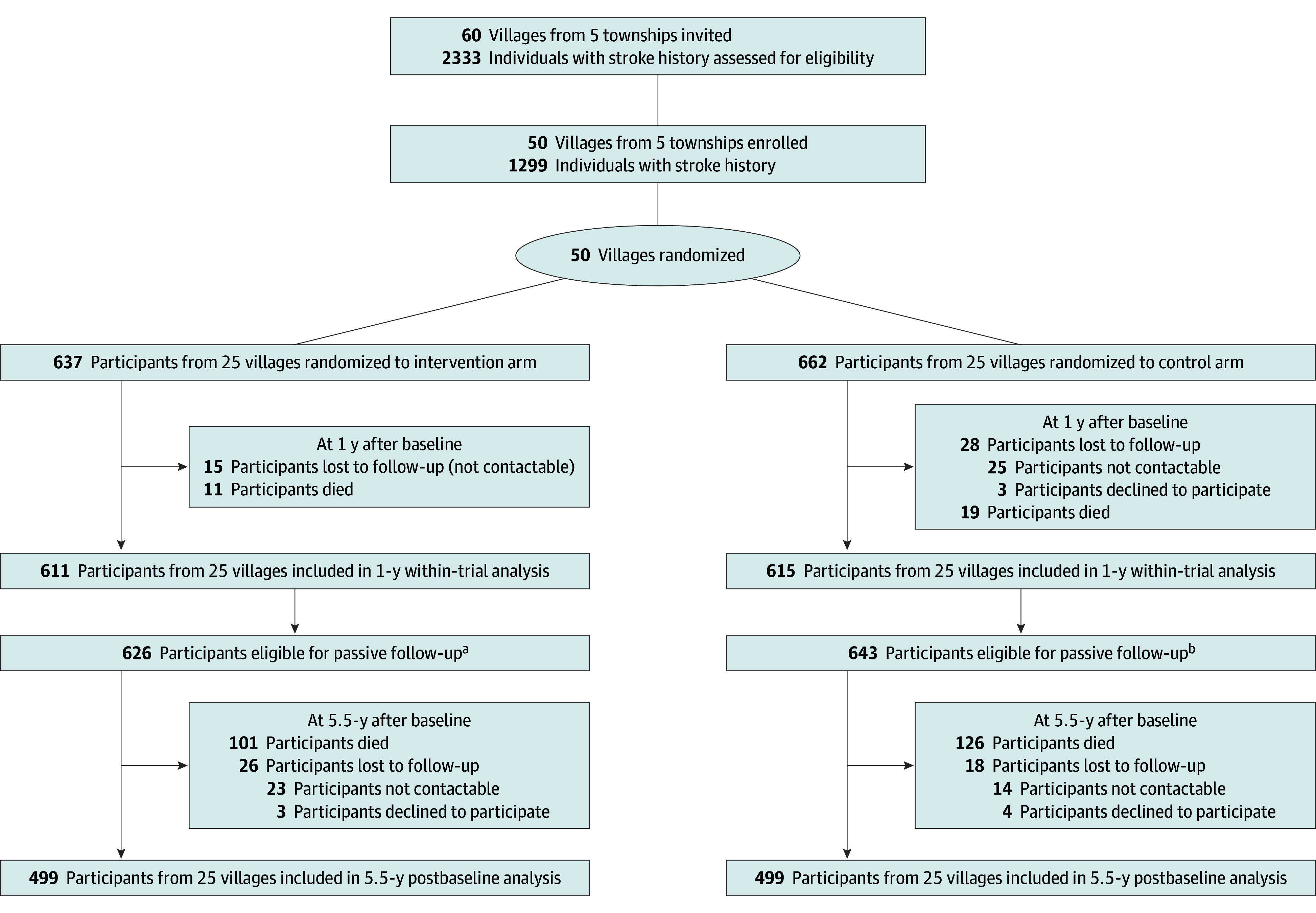
Trial Flowchart, 2017 to 2023 At baseline, the intervention arm included a mean (SD) of 25.5 (3.2) villages; the control arm, 26.5 (2.7) villages. At 1-year follow-up, the intervention arm included a mean (SD) of 24.4 (3.2) villages; the control arm, 24.6 (2.8) villages. At the 5.5-year follow-up, the intervention arm included a mean (SD) of 20.0 (3.2) villages; the control arm, 20.0 (3.0) villages. ^a^Includes those alive at 12 months after baseline (ie, including both 611 participants who were followed up and 15 participants who were lost to follow-up at 12 months). ^b^Includes those alive at 12 months after baseline (ie, including 615 participants who were followed up and 28 who were lost to follow-up at 12 months).

Among 1299 participants recruited at baseline, 30 died and 43 were lost to follow-up by the 1-year assessment, so that 1226 people completed the assessment (Figure 1). Another 227 participants died during the long-term observational follow-up period. Of 1042 surviving participants eligible for the 5.5-year follow up, 998 completed the assessment (44 [4.2%] were lost to follow-up, consisting of 26 [2.5%] in the intervention arm and 18 [1.7%] in the control arm), including 640 who completed in October 2022, 339 in May 2023, and 19 in August 2023, with a mean (SD) duration of 66.6 (3.7) months (66.2 [3.5] months after baseline for the intervention arm and 67.0 [3.9] months after baseline for the control arm) (eFigure 1 in Supplement 2).

Among the 998 participants at the 5.5-year follow-up, baseline characteristics remained well balanced between intervention and control arms (Table 1), with 454 (45.5%) female and 544 (54.5%) male. Most participants were older adults (mean age, 65.0 [8.2] years at baseline) and had no formal education (423 [42.4%]), and almost half had an annual household income below ¥5000 (476 of 991 with data available [48.0%]) (¥1 = US $0.14). Most participants (694 [69.5%]) reported hypertension at baseline. We observed differences between those completing the posttrial assessment and those who were either lost to follow-up (n = 44) or deceased (n = 257) in age, sex, lifestyle, quality of life, and disease history (eTable 1 in Supplement 2). Among participants who completed the posttrial follow-up, changes in marital status, disease history, and smartphone use were observed from baseline to 5.5 years (eTable 2 in Supplement 2).

**Table 1.** Baseline Characteristics of Participants at the 5.5-Year Follow-Up

Characteristic	Trial arm, No. (%)^a^		
	Intervention (n = 499)	Control (n = 499)	All (n = 998)
Demographic and lifestyle			
Age, mean (SD), y	65.6 (8.1)	64.4 (8.2)	65.0 (8.2)
Sex			
Female	231 (46.3)	223 (44.7)	454 (45.5)
Male	268 (53.7)	276 (55.3)	544 (54.5)
Educational attainment			
No schooling	217 (43.5)	206 (41.3)	423 (42.4)
Some schooling or primary school	140 (28.1)	158 (31.7)	298 (29.9)
Secondary school and above	142 (28.5)	135 (27.1)	277 (27.8)
Marital status			
Married	408 (81.8)	424 (85.0)	832 (83.4)
Widowed, divorced, or not married	91 (18.2)	75 (15.0)	166 (16.6)
Annual household income^b^			
<¥5000	230 (46.5)	246 (49.6)	476 (48.0)
≥¥5000	265 (53.5)	250 (50.4)	515 (52.0)
Phone ownership			
No phone (may have a shared phone)	119 (23.8)	100 (20.0)	219 (21.9)
Basic phone	353 (70.7)	348 (69.7)	701 (70.2)
Smartphone	27 (5.4)	51 (10.2)	78 (7.8)
Had none of the listed assets^c^	23 (4.6)	39 (7.8)	62 (6.2)
Smoking status			
Current	75 (15.0)	83 (16.6)	158 (15.8)
Former	90 (18.0)	97 (19.4)	187 (18.7)
Never	334 (66.9)	319 (63.9)	653 (65.4)
Stroke history			
Ischemic	436 (87.4)	424 (85.0)	860 (86.2)
Hemorrhagic	61 (12.2)	74 (14.8)	135 (13.5)
Not specified	2 (0.4)	1 (0.2)	3 (0.3)
Stroke duration, median (IQR), y			
Since the first event	5.00 (2.00-10.00)	5.00 (2.00-10.00)	5.00 (2.00-10.00)
Since the latest event^d^	3.00 (1.00-7.00)	3.00 (1.00-7.00)	3.00 (1.00-7.00)
Self-reported diseases			
Hypertension	357 (71.5)	337 (67.5)	694 (69.5)
Dyslipidemia	192 (38.5)	208 (41.7)	400 (40.1)
Type 1 or 2 diabetes	85 (17.0)	75 (15.0)	160 (16.0)
Heart disease	49 (9.8)	42 (8.4)	91 (9.1)
Outcomes at baseline			
Systolic blood pressure, mean (SD), mm Hg	146.0 (21.1)	146.1 (22.4)	146.1 (21.7)
Diastolic blood pressure, mean (SD), mm Hg	78.3 (11.8)	80.1 (11.4)	79.2 (11.6)
Taking antihypertensive medications	408 (81.8)	388 (77.8)	796 (79.8)
Adherence to antihypertensive medication regimen^e^	259 (63.5)	237 (61.1)	496 (62.3)
Moderate to severe disability^f^	134 (26.9)	113 (22.6)	247 (24.7)
Stroke recurrence since diagnosis	129 (25.9)	142 (28.5)	271 (27.2)

^a^ Percentages have been rounded and may not total 100.

^b^ ¥1 = US $0.14. Missing for 4 participants in the intervention arm and 3 in the control arm.

^c^ Includes television, refrigerator, air conditioner, and computer.

^d^ Missing data for 2 participants in the intervention arm and 1 in the control arm.

^e^ Measured among participants who were using the medicine (408 and 388 in intervention and control arms, respectively) based on the 4-item Morisky Green Levine Scale (scored from 0 to 4, with lower scores indicating higher adherence); people who received a score of 0 were defined as having adherence to the regimen.

^f^ Measured by the modified Rankin Scale, with scores ranging from 0 (no symptoms) to 5 (severe disability); people with a score of 3 or higher were categorized into the "moderate to severe disability" group.

**Figure 2.**
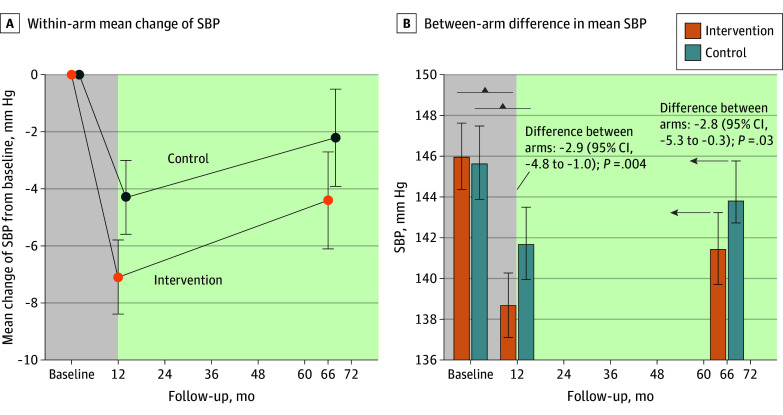
Long-Term Systolic Blood Pressure (SBP) From Baseline to the 1- and 5.5-Year Follow-Ups The lightly shaded area indicates 1-year active intervention period. More dark shading indicates observational follow-up after the trial ended. Error bars indicate 95% CIs. In B, the 2 small arrows to the right indicate that the difference shown is from baseline to follow-up.

At 5.5 years, the intervention arm had a model-estimated mean reduction in systolic BP of 4.4 (95% CI, 2.7-6.1) mm Hg from baseline, attenuated from the reduction of 7.1 (95% CI, 5.8-8.4) mm Hg observed at the 1-year follow-up (Figure 2A). The control arm had a model-estimated reduction of 2.2 (95% CI, 0.5-3.9) mm Hg from baseline to 5.5 years, attenuated from the reduction of 4.3 (95% CI, 3.0-5.6) mm Hg and leading to a significant between-arm net difference of −2.8 (95% CI, −5.3 to −0.3) mm Hg (*P* = .03; intraclass correlation <0.001) at 5.5 years, slightly smaller than that at 1 year (−2.9 [95% CI, −4.8 to −1.0] mm Hg; *P* = .004) (Figure 2B).

**Figure 3.**
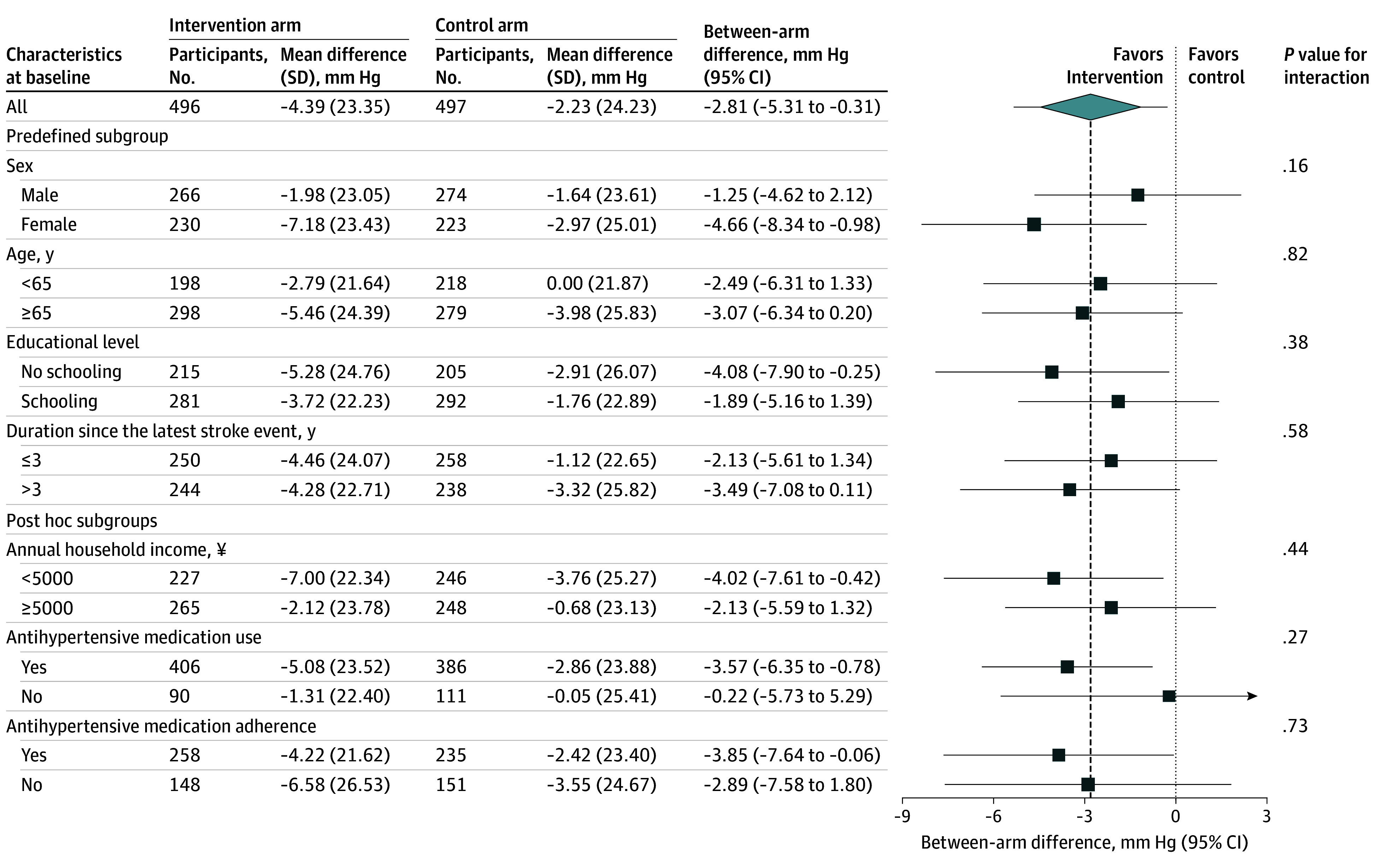
Mean Adjusted Differences in Systolic Blood Pressure (SBP) Change From Baseline to 5.5-Year Follow-Up by Baseline Subgroup The dotted line represents no effect, and the dashed line represents the overall estimated effect. Three SBP outliers were removed from the intervention arm and 2 from the control arm. Subgroup medication adherence was only measured among participants who were taking the medicine at baseline (406 in the intervention arm and 386 in the control arm).

The effect of the intervention did not significantly differ between any of the subgroups. However, in line with the overall effect, significant between-arm differences (ie, the 95% CIs do not include 0) were observed among female participants, individuals with no formal schooling, those with lower annual household income, and those who used and adhered to antihypertensive medications at baseline (Figure 3). We also observed significant between-arm differences among those having a baseline self-reported hypertension, those whose baseline systolic BP was 140 mm Hg or higher, and those having not experienced stroke recurrence since diagnosis (eFigure 2 in Supplement 2).

The analysis of secondary outcomes revealed a sustained reduction in nonfatal stroke recurrence risk in the intervention arm (RR, 0.77; 95% CI, 0.61-0.99) compared with controls (Table 2), and a lower absolute risk of stroke recurrence (6.0 [95% CI, −11.3 to −0.7] percentage points) (eTable 3 in Supplement 2). Other metrics such as controlled systolic BP also demonstrated a positive, though decreased, difference favoring the intervention arm.

**Table 2.** Within-Trial and Long-Term Additional Outcomes^a^

Outcome	1-y Within-trial assessment^b^			5.5-y Postbaseline assessment^b^		
	Trial arm		Mean difference or RR (95% CI)	Trial arm		Mean difference or RR (95% CI)
	Intervention (n = 611)	Control (n = 615)		Intervention (n = 499)	Control (n = 499)	
Continuous, mean (SD), mm Hg						
Change in SBP	−7.1 (18.5)	−4.3 (18.9)	−2.9 (−4.8 to −1.0)	−4.4 (23.3)	−2.2 (24.2)	−2.8 (−5.3 to −0.3)
Change in DBP	−3.9 (9.6)	−2.3 (9.6)	−2.2 (−3.2 to −1.2)	3.1 (11.4)	3.4 (11.1)	−1.1 (−2.4 to 0.2)
Binary, No. (%)						
Controlled SBP^c^	335 (55.3)	296 (48.4)	1.16 (1.05 to 1.27)	243 (49.0)	220 (44.3)	1.13 (1.01 to 1.27)
Controlled BP^d^	330 (54.3)	281 (45.9)	1.20 (1.09 to 1.32)	220 (44.3)	204 (41.4)	1.09 (0.97 to 1.22)
Medication use and adherence						
Taking antihypertensive medications	521 (85.3)	475 (77.2)	1.06 (1.01 to 1.11)	434 (87.0)	410 (82.2)	1.03 (0.98 to 1.07)
Adherence to antihypertensive medication regimen^e^	383 (73.5)	315 (66.3)	1.10 (1.00 to 1.22)	290 (66.8)	249 (60.7)	1.11 (1.00 to 1.23)
Disability and stroke recurrence						
Moderate to severe disability^f^	128 (20.9)	186 (30.2)	0.64 (0.53 to 0.79)	165 (33.1)	165 (33.1)	0.93 (0.78 to 1.10)
Stroke recurrence^g^	27 (4.4)	57 (9.3)	0.42 (0.26 to 0.68)	101 (20.2)	128 (25.7)	0.77 (0.61 to 0.99)

Abbreviations: DBP, diastolic blood pressure (BP); RR, risk ratio; SBP, systolic blood pressure.

^a^ Mixed-effects linear regression models were used for continuous variables (mean difference), and generalized estimating equations were used for binary outcomes (RR [95% CI]).

^b^ Adjusted for baseline outcome, township, sex, age, and month of interview.

^c^ Defined as less than 140 mm Hg. Five participants for the intervention and 4 for the control arms were excluded as outliers at 1 year; 3 and 2, respectively, were excluded as outliers at 5.5 years.

^d^ Defined as SBP of less than 140 mm Hg and DBP of less than 90 mm Hg. Three participants for the intervention and 3 for the control arms were excluded as outliers at 1 year; 2 and 6, respectively, were excluded as outliers at 5.5 years.

^e^ Measured among participants who were using medicines, based on the 4-item Morisky Green Levine Scale (scored from 0 to 4, with lower scores indicating higher adherence); people who received a score of 0 were defined as having adherence to the regimen. Medication adherence outcomes were not adjusted for baseline outcome, since the set of participants taking medication at baseline was not the same set taking the medicine at follow-up.

^f^ Measured by the modified Rankin Scale, with scores ranging from 0 (no symptoms) to 5 (severe disability); people with a score of 3 or higher were categorized into the "moderate to severe disability" group.

^g^ Stroke recurrence refers to any recurrent stroke 1 year after baseline and any recurrent stroke in 5.5 years after baseline. The generalized estimating equations model for 1-year within-trial assessment did not converge after we additionally adjusted month of interview, so we used a marginal standardization approach by fitting a binomial model with a logit link to obtain the RR.

We conducted sensitivity analyses including models adjusted for posttrial follow-up duration, baseline imbalance, and imbalance related to loss to follow-up and death (eTable 4 in Supplement 2); models excluding those who did not have all 3 assessments (eTable 5 in Supplement 2); and models excluding individuals who completed posttrial assessment in the summer (eTable 6 in Supplement 2). Results were generally consistent. For example, the estimated between-arm difference in change in systolic BP ranged from −2.8 to −3.1 mm Hg across all models, while the RR for stroke recurrence ranged from 0.76 (95% CI, 0.60-0.96) to 0.78 (95% CI, 0.61-0.99).

## Discussion

Upon concluding the 1-year SINEMA intervention and 5.5-year follow-up of 998 patients who survived stroke in rural China, this secondary analysis of a cRCT found a modest but significant between-arm net decrease in systolic BP (−2.8 mm Hg). Most secondary outcomes showed sustained effects, with a notable absolute decrease of 6.0 percentage points in stroke recurrence (RR, 0.77). Significant between-arm differences were observed among women, people with lower educational attainment, lower income, and higher use of and adherence to antihypertensive medications at baseline. These findings, alongside the primary 1-year trial results,^11^ substantiate the short-term and long-term benefits of the SINEMA intervention and its impact in diminishing health inequity across urban-rural, gender, and socioeconomic lines.

Our study is innovative and groundbreaking in the following 4 ways. First, it is one of a few studies that have examined the long-term outcome of primary care–based interventions on BP control.^30,31,32,33,34^ Previous long-term studies showed mixed findings: the COBIN (Community-Based Management of Hypertension in Nepal) trial reported an unexpected potential harm with an increase of 4.1 mm Hg in the intervention than control arm at 5 years^31^; a home BP telemonitoring program found no difference at 54 months^34^; and the COBRA (Control of Blood Pressure and Risk Attenuation) trial found more reduction in the intervention arm (between-arm difference: −2.1 mm Hg) at 7 years.^30^ Our study that is, to our knowledge, the first cRCT with long-term follow-up among patients with stroke, found a slightly attenuated yet sustained benefit. The magnitude of the long-term systolic BP reduction in our study was similar to that found in the COBRA trial but much smaller than in the China Rural Hypertension Control Project (−14.5 mm Hg at 18 months within trial),^6,35^ which recruited people with hypertension and, more importantly, provided free medicine to most participants. Moreover, we found that the long-term benefit in systolic BP was more pronounced in patients using antihypertensive medications at baseline and adhering to the regimen, compared with nonusers or nonadherent users. These results, together with the findings on the lasting effects of the SINEMA intervention on medication adherence, highlight the critical roles of medication use and adherence in stroke management.

Second, our study demonstrated sustained benefits not only for BP but also stroke recurrence among this high-risk population with existing stroke. We observed 23% relative and 6% absolute reduction in stroke recurrence. The underlining mechanisms of lowering stroke risks with the SINEMA intervention are multidimensional, resulting from the multiple components of the SINEMA package that targeted the control of multiple risk factors, including systolic BP as well as statin and aspirin use and physical activity. Our study is not able to fully reveal all the potential mechanisms of change, but evidence^36^ suggests that even a 1-mm Hg reduction in systolic BP could lead to a 5% reduction in risk of stroke and meaningful decrease in cardiovascular risks, highlighting the importance of BP control among patients who survive stroke. Our findings on the reduction of stroke recurrence hold significant clinical and public health implications, given the increasing burden of stroke in China and globally,^14^ along with the high risk of stroke recurrence and death among patients with a previous stroke.^37,38^

Third, our study has important implications for reducing urban-rural disparities and potentially health inequity by socioeconomic status. In China, the incidence, prevalence, and mortality of stroke in rural China exceeded those in urban areas,^39^ while the awareness, treatment, and control of hypertension and stroke were lower among patients from rural areas.^39,40,41,42^ The SINEMA intervention with long-term health benefits for rural patients can thus contribute to narrowing the urban-rural gap. In rural areas, women and individuals with lower socioeconomic status usually exhibit a lower level of awareness and knowledge of stroke management^43^ and use fewer health services due to availability and affordability issues.^40,41,44^ Thus, it is plausible that these vulnerable groups may benefit more from the primary care–based intervention in increasing access and quality of care in their communities. The SINEMA intervention holds promise in mitigating health inequity related to gender and socioeconomic status.

Last, several key features of the SINEMA intervention designed with sustainability from the outset^16^ help to explain the sustained effects we observed.^17^ First, this management program, informed by robust evidence and contextualized to resource-limited settings, integrates clinical guidelines,^45^ the chronic care model,^19^ and extensive field research,^17,21,22^ ensuring its sustainability.^46,47,48,49^ Second, using existing health human resources—village doctors in the context of rural China—means the likelihood of posttrial sustainability is higher. For village doctors, main strategies included training, nonfinancial incentives such as recognition and awards, and performance-based financial incentives, with the effects of the first two expected to persist beyond the trial’s conclusion.^17^ Third, mobile health technology was crucial to our intervention.^30,31,34,50,51,52^ The SINEMA app, featuring user-friendly functions such as reminders and BP tracking, free for village doctors, could be used after the trial stopped.^21^ Although the free voice messages to patients were not dispatched after the active trial intervention, the practical tips and reinforcing recommendations may change participants’ behavior in the long run.^22^ The values of mobile health for noncommunicable disease management have been demonstrated by many systematic reviews.^53,54,55,56^ These 3 features are not only relevant for stroke management in rural China, but potentially transferable and adaptable to other conditions and countries facing similar challenges.

### Limitations

Our study’s limitations merit consideration. While we identified key indicators such as medication adherence as possible explanations of observed benefits, the cRCT design could not attribute effects to specific components or enable explanations of change mechanisms. However, we plan to conduct qualitative research to enrich our understanding in these areas. In addition, some other important factors for BP and stroke, such as salt intake and other dietary patterns, were not targeted in our intervention design and were not measured. However, the design of our complex health intervention containing multiple components was informed and based on extensive contextual research for feasibility and cost-effectiveness.^16,22,57^ In addition, the study was conducted in a single county in Northern China, limiting its generalizability. Future research is planned to identify transferrable and adaptable strategies to other settings. Due to COVID-19 disruptions, we were not able to conduct multiple follow-ups, and our recent follow-up could not be completed during a single period. Despite these challenges, data collection and between-arm participant characteristics remained balanced. We also conducted sensitivity analyses that yielded robust results. Stroke recurrence was measured based on self-reports of nonfatal recurrence events with care seeking in the county hospital and above. Accurate information on the exact event dates, severity of the events, and imaging evidence were not available. Some patients may mistakenly report nonstroke symptoms, which may lead to the overestimation of stroke recurrence, while others may fail to report past recurrent strokes, leading to underestimation. To address these common problems from self-reported data, linkage with electronic health records and insurance claims is planned for additional analyses in the future.

## Conclusions

This secondary analysis of a cRCT found that a 1-year primary care–based intervention for stroke management was associated with reduced systolic BP and stroke recurrence over 4.5 years after the active intervention ceased. These results of sustained changes are clinically significant, especially for secondary stroke prevention in areas with limited resources. They also provide evidence for scaling up of the intervention, and discussions with policymakers in China are ongoing. Future research should pinpoint implementation strategies essential for the intervention’s sustainability and transferability, aiding its expansion and contextual adaptation in China and other low- and middle-income countries.

## Notes

Supplement 1.
Trial Protocol

Supplement 2. **eTable 1.** Baseline Characteristics by Treatment Arm and 5.5-Year Follow-Up Status **eTable 2.** Participant Characteristics at Baseline and at the 5.5-Year Follow-Up **eTable 3.** Within-Trial and Long-Term Secondary Outcomes Reported as Risk Differences (Percentage Points) **eTable 4.** Additional Sensitivity Analyses **eTable 5.** Sensitivity Analyses of Within-Trial and Long-Term Outcomes Among 969 Participants Who Completed All 3 Assessments **eTable 6.** Sensitivity Analysis of Long-Term Outcomes by Excluding 19 Participants Who Completed the 5.5-Year Post-Baseline Assessment in August 2023 **eFigure 1.** Timeline and Follow-Up Duration of the SINEMA Main Trial and Post-Trial **eFigure 2.** Mean Adjusted Differences in Systolic Blood Pressure Change From Baseline to 5.5-Year Follow-Up by Baseline Subgroup

Supplement 3.
Data Sharing Statement
