# Supplementary material for: Primary Care–Based Digital Health–Enabled Stroke Management Intervention: Long-Term Follow-Up of a Cluster Randomized Clinical Trial
Source: JAMA Netw Open. 2024 Dec 13;7(12):e2449561. doi: 10.1001/jamanetworkopen.2024.49561 (PMC11645652; doi:10.1001/jamanetworkopen.2024.49561)
Supplement: Supplement 2. — eTable 1. Baseline Characteristics by Treatment Arm and 5.5-Year Follow-Up Status eTable 2. Participant Characteristics at Baseline and at the 5.5-Year Follow-Up eTable 3. Within-Trial and Long-Term Secondary Outcomes Reported as Risk Differences (Percentage Points) eTable 4. Additional Sensitivity Analyses eTable 5. Sensitivity Analyses of Within-Trial and Long-Term Outcomes Among 969 Participants Who Completed All 3 Assessments eTable 6. Sensitivity Analysis of Long-Term Outcomes by Excluding 19 Participants Who Completed the 5.5-Year Post-Baseline Assessment in August 2023 eFigure 1. Timeline and Follow-Up Duration of the SINEMA Main Trial and Post-Trial eFigure 2. Mean Adjusted Differences in Systolic Blood Pressure Change From Baseline to 5.5-Year Follow-Up by Baseline Subgroup [file jamanetwopen-e2449561-s002.pdf]

Supplementary Online Content

Tan J, Gong E, Gallis JA, et al. Primary care–based digital health–enabled stroke management intervention: long-term follow-up of a cluster randomized clinical trial. *JAMA Netw Open*. 2024;7(12):e2449561. doi:10.1001/jamanetworkopen.2024.49561

eTable 1. Baseline Characteristics by Treatment Arm and 5.5-Year Follow-Up Status
eTable 2. Participant Characteristics at Baseline and at the 5.5-Year Follow-Up
eTable 3. Within-Trial and Long-Term Secondary Outcomes Reported as Risk Differences (Percentage Points)
eTable 4. Additional Sensitivity Analyses
eTable 5. Sensitivity Analyses of Within-Trial and Long-Term Outcomes Among 969 Participants Who Completed All 3 Assessments
eTable 6. Sensitivity Analysis of Long-Term Outcomes by Excluding 19 Participants Who Completed the 5.5-Year Post-Baseline Assessment in August 2023
eFigure 1. Timeline and Follow-Up Duration of the SINEMA Main Trial and Post-Trial
eFigure 2. Mean Adjusted Differences in Systolic Blood Pressure Change From Baseline to 5.5-Year Follow-Up by Baseline Subgroup

This supplementary material has been provided by the authors to give readers additional information about their work.

© 2024 Tan J et al. *JAMA Network Open*.

eTable 1. Baseline Characteristics by Treatment Arm and 5.5-Year Follow-Up Status*

Characteristics, n (%)	Baseline assessment		5.5-year post-baseline assessment			Overall (n=1299)
	Intervention (n=637)	Control (n=662)	Followed-up (n=998)	Died (n=257)	Loss to follow-up (n=44)	
**Demographic characteristics and lifestyle**						
**Age, years, mean (SD)**	66.2 (8.2)	65.2 (8.2)	65.0 (8.2)	69.0 (7.2)	62.1 (8.8)	65.7 (8.2)
**Sex, female**	272 (42.7)	281 (42.4)	454 (45.5)	81 (31.5)	18 (40.9)	553 (42.6)
**Education**						
No schooling	264 (41.4)	274 (41.4)	423 (42.4)	100 (38.9)	15 (34.1)	538 (41.4)
Some schooling or primary school	182 (28.6)	205 (31.0)	298 (29.9)	76 (29.6)	13 (29.5)	387 (29.8)
Secondary school or above	191 (30.0)	183 (27.6)	277 (27.8)	81 (31.5)	16 (36.4)	374 (28.8)
**Marital status**						
Married	526 (82.6)	549 (82.9)	832 (83.4)	206 (80.2)	37 (84.1)	1075 (82.8)
Widowed, divorced, or not married	111 (17.4)	113 (17.1)	166 (16.6)	51 (19.8)	7 (15.9)	224 (17.2)
**Annual household income, Chinese Yuan**						
<5,000	293 (46.3)	334 (50.8)	476 (48.0)	132 (51.6)	19 (43.2)	627 (48.6)
≥5,000	340 (53.7)	324 (49.2)	515 (52.0)	124 (48.4)	25 (56.8)	664 (51.4)
**Phone ownership**						
No phone (may have a shared phone)	164 (25.7)	159 (24.0)	219 (21.9)	96 (37.4)	8 (18.2)	323 (24.9)
Basic phone	435 (68.3)	440 (66.5)	701 (70.2)	149 (58.0)	25 (56.8)	875 (67.4)
Smartphone	38 (6.0)	63 (9.5)	78 (7.8)	12 (4.7)	11 (25.0)	101 (7.8)
**Phone sharing**						
No phone (may have a shared phone)	164 (25.7)	159 (24.0)	219 (21.9)	96 (37.4)	8 (18.2)	323 (24.9)
Sharing with family	187 (29.4)	212 (32.0)	316 (31.7)	71 (27.6)	12 (27.3)	399 (30.7)

© 2024 Tan J et al. *JAMA Network Open*.

Characteristics	Baseline assessment		5.5-year post-baseline assessment			Overall (n=1299)
	Intervention (n=637)	Control (n=662)	Followed-up (n=998)	Died (n=257)	Loss to follow-up (n=44)	
Owning a phone	286 (44.9)	291 (44.0)	463 (46.4)	90 (35.0)	24 (54.5)	577 (44.4)
**Had none of the listed assets**[a]	28 (4.4)	50 (7.6)	62 (6.2)	15 (5.8)	1 (2.3)	78 (6.0)
**Smoking status**						
Current smoker	99 (15.5)	122 (18.4)	158 (15.8)	50 (19.5)	13 (29.5)	221 (17.0)
Former smoker	130 (20.4)	132 (19.9)	187 (18.7)	67 (26.1)	8 (18.2)	262 (20.2)
Never smoker	408 (64.1)	408 (61.6)	653 (65.4)	140 (54.5)	23 (52.3)	816 (62.8)
**Disease history**						
**Stroke type**						
Ischemic stroke	555 (87.1)	564 (85.2)	860 (86.2)	225 (87.5)	34 (77.3)	1119 (86.1)
Hemorrhage stroke	80 (12.6)	96 (14.5)	135 (13.5)	31 (12.1)	10 (22.7)	176 (13.5)
Not specified	2 (0.3)	2 (0.3)	3 (0.3)	1 (0.4)	0 (0.0)	4 (0.3)
**Stroke duration, years, median (IQR)**						
Since the first event	5.00 (2.00-10.00)	5.00 (2.00-10.00)	5.00 (2.00-10.00)	6.00 (3.00-11.00)	6.50 (3.00-10.50)	5.00 (2.00-10.00)
Since the latest event[b]	3.00 (1.00-7.00)	3.00 (1.00-7.00)	3.00 (1.00-7.00)	3.00 (1.00-7.00)	3.50 (1.50-7.00)	3.00 (1.00-7.00)
**Self-reported diseases**						
Hypertension	461 (72.4)	436 (65.9)	694 (69.5)	170 (66.1)	33 (75.0)	897 (69.1)
Dyslipidemia	248 (38.9)	271 (40.9)	400 (40.1)	101 (39.3)	18 (40.9)	519 (40.0)
Diabetes	113 (17.7)	103 (15.6)	160 (16.0)	48 (18.7)	8 (18.2)	216 (16.6)
Heart disease	70 (11.0)	54 (8.2)	91 (9.1)	28 (10.9)	5 (11.4)	124 (9.5)

© 2024 Tan J et al. *JAMA Network Open*.

Characteristics	Baseline assessment		5.5-year post-baseline assessment			Overall (n=1299)
	Intervention (n=637)	Control (n=662)	Followed-up (n=998)	Died (n=257)	Loss to follow-up (n=44)	
Depression[c]	61 (9.6)	48 (7.3)	72 (7.2)	33 (12.8)	4 (9.1)	109 (8.4)
**Physical activity, functioning, and quality of life**						
Achieve health enhancing physical activity[d]	99 (15.5)	114 (17.2)	178 (17.8)	23 (8.9)	12 (27.3)	213 (16.4)
Completion time of timed up and go test, second, median (IQR)[e]	14.10 (11.60-19.55)	14.30 (11.50-20.00)	13.80 (11.30-18.10)	18.00 (13.00-25.90)	12.65 (10.57-19.10)	14.20 (11.50-19.90)
Body Mass Index, kg/m², mean (SD)[f]	25.5 (3.7)	25.5 (3.6)	25.8 (3.6)	24.3 (3.7)	26.2 (3.3)	25.5 (3.7)
Health-related quality of life in utility, mean (SD)[g]	0.8 (0.2)	0.8 (0.2)	0.8 (0.2)	0.7 (0.3)	0.9 (0.2)	0.8 (0.2)
**Outcomes at baseline**						
Systolic blood pressure, mmHg, mean (SD)	146.0 (20.9)	145.7 (23.7)	146.1 (21.7)	145.2 (24.5)	145.0 (24.2)	145.9 (22.4)
Diastolic blood pressure, mmHg, mean (SD)	78.0 (11.6)	79.7 (11.7)	79.2 (11.6)	77.5 (12.1)	80.0 (10.5)	78.9 (11.7)
Taking antihypertensive medications	522 (81.9)	508 (76.7)	796 (79.8)	200 (77.8)	34 (77.3)	1030 (79.3)
Adherence to antihypertensive medications[h]	329 (63.0)	316 (62.2)	496 (62.3)	128 (64.0)	21 (61.8)	645 (62.6)
Moderate to severe disability[i]	179 (28.1)	173 (26.1)	247 (24.8)	99 (38.5)	6 (13.6)	352 (27.1)
Stroke recurrence since diagnosis	178 (27.9)	200 (30.2)	271 (27.2)	94 (36.6)	13 (29.5)	378 (29.1)

*Data are n (%) except where described as mean (standard deviation, SD), or median (interquartile range; *IQR*). The baseline assessment characteristics overall and separately for intervention and control arms are reported herein for context but were published before.

[a] TV, refrigerator, air conditioner, and computer were listed as home assets in the questionnaire.

[b] For baseline assessment, three participants are missing data in intervention arm and two are missing data in control arm; and for 5.5-year post-baseline assessment, three participants are missing data among follow-up participants, two are missing data among deceased participants.

[c] Depression was measured by the Patient Health Questionnaire-2, and people who received a score above 2 were defined as the depression group.

[d] Health Enhancing Physical Activity: a) vigorous-intensity activity on at least 3 days achieving a minimum of at least 1500 MET-minutes/week OR b) 7 or more days of any combination of walking, moderate-intensity or vigorous intensity activities achieving a minimum of at least 3000 MET-

© 2024 Tan J et al. *JAMA Network Open*.

minutes/week.

[e] For baseline assessment, nine participants are missing data in intervention arm and nine are missing data in control arm; and for 5.5-year post-baseline assessment, eight participants are missing data among follow-up participants, 10 are missing data among deceased participants.

[f] For baseline assessment, six participants are missing data in intervention arm and five are missing data in control arm; and for 5.5-year post-baseline assessment, four participants are missing data among follow-up participants, seven are missing data among deceased participants.

[g] Health-related quality of life was measured by using EQ5D-5L and was converted into a utility score based on the Chinese value set. The utility score ranged from −0.4 to 1.

[h] Medication adherence was only measured among participants who were taking the medicine based on 4-item Morisky Green Levine Scale, and people who received a score of 0 were defined as the “adherence to antihypertensive medications” group.

[i] Disability was measured by the modified Rankin Scale, with scores ranging from 0 (no symptoms) to 5 (severe disability); people with a score of ≥3 were categorized into the "moderate to severe disability" group.

© 2024 Tan J et al. *JAMA Network Open*.

eTable 2. Participant Characteristics at Baseline and at the 5.5-Year Follow-Up*

Characteristics, n (%)	Baseline assessment			5.5-year post-baseline assessment		
	Intervention (n=499)	Control (n=499)	Total (n=998)	Intervention (n=499)	Control (n=499)	Total (n=998)
**Demographic characteristics and lifestyle**						
**Marital status**						
Married	408 (81.8)	424 (85.0)	832 (83.4)	377 (75.6)	396 (79.4)	773 (77.5)
Widowed, divorced, or not married	91 (18.2)	75 (15.0)	166 (16.6)	122 (24.4)	103 (20.6)	225 (22.5)
**Annual household income, Chinese Yuan**						
<5,000	230 (46.5)	246 (49.6)	476 (48.0)	196 (42.5)	150 (32.8)	346 (37.7)
≥5,000	265 (53.5)	250 (50.4)	515 (52.0)	265 (57.5)	307 (67.2)	572 (62.3)
**Phone ownership**						
No phone (may have a shared phone)	119 (23.8)	100 (20.0)	219 (21.9)	116 (23.2)	123 (24.6)	239 (23.9)
Basic phone	353 (70.7)	348 (69.7)	701 (70.2)	271 (54.3)	252 (50.5)	523 (52.4)
Smartphone	27 (5.4)	51 (10.2)	78 (7.8)	112 (22.4)	124 (24.8)	236 (23.6)
**Smoking status**						
Current smoker	75 (15.0)	83 (16.6)	158 (15.8)	68 (13.6)	80 (16.0)	148 (14.8)
Former smoker	90 (18.0)	97 (19.4)	187 (18.7)	107 (21.4)	108 (21.6)	215 (21.5)
Never smoker	334 (66.9)	319 (63.9)	653 (65.4)	324 (64.9)	311 (62.3)	635 (63.6)
**Self-reported diseases**						
Hypertension	357 (71.5)	337 (67.5)	694 (69.5)	438 (87.8)	432 (86.6)	870 (87.2)
Dyslipidemia	192 (38.5)	208 (41.7)	400 (40.1)	243 (48.7)	264 (52.9)	507 (50.8)
Diabetes	85 (17.0)	75 (15.0)	160 (16.0)	102 (20.4)	106 (21.2)	208 (20.8)
Heart disease	49 (9.8)	42 (8.4)	91 (9.1)	59 (11.8)	55 (11.0)	114 (11.4)

* Data are n (%). Baseline assessment characteristics as in Table 1, reported for context.

© 2024 Tan J et al. *JAMA Network Open*.

eTable 3. Within-Trial and Long-Term Secondary Outcomes Reported as Risk Differences (Percentage Points)[a]

	1-year within-trial assessment			5.5-year post-baseline assessment		
Outcomes, n (%)	**Intervention (n=611)**	**Control (n=615)**	**Risk difference (95% CI)**	**Intervention (n=499)**	**Control (n=499)**	**Risk difference (95% CI)**
**Blood pressure related outcomes[b]**						
Controlled systolic BP[c]	335 (55.3)	296 (48.5)	8.1pp (3.1 to 13.1)	243 (49.0)	220 (44.3)	7.1pp (2.2 to 12.1)
Controlled BP[d]	330 (54.7)	281 (46.2)	10.2pp (5.1 to 15.2)	220 (44.4)	204 (41.4)	4.8pp (0.1 to 9.5)
**Medication use and adherence**						
Taking antihypertensive medications	521 (85.3)	475 (77.2)	5.1pp (-0.5 to 10.8)	434 (87.0)	410 (82.2)	2.7pp (-2.3 to 7.7)
Adherence to antihypertensive medications[e],	383 (73.7)	315 (66.5)	6.9pp (-0.1 to 13.9)	290 (66.8)	249 (60.7)	6.6pp (0.1 to 13.0)
**Disability and stroke recurrence**						
Moderate to severe disability[f]	128 (21.0)	186 (30.2)	-10.8pp (-15.2 to -6.4)	165 (33.1)	165 (33.1)	-2.1pp (-7.4 to 3.1)
Stroke recurrence[g]	27 (4.4)	57 (9.3)	-5.2pp (-8.0 to -2.4)	101 (20.7)	128 (26.8)	-6.0pp (-11.3 to -0.7)

Abbreviations: CI, confidence interval; BP, blood pressure.

[a] Adjusted for baseline outcome, township, sex, age, and month of interview, and removing outliers in systolic and diastolic blood pressure based on an *a priori* decision to remove those that are more than 2 interquartile range above the third quartile or below the first quartile.

[b] The numbers and proportions of controlled systolic blood pressure and controlled blood pressure also removed outliers. Systolic blood pressure outliers were removed resulting in 5 and 4 removed from intervention and control arms, respectively, at 1-year follow-up; and 3 and 2 removed from intervention and control arms, respectively, at 5.5-year follow-up. Diastolic blood pressure outliers were removed resulting in 3 and 3 removed from intervention and control arms, respectively, at 1-year follow-up; and 2 and 6 removed from intervention and control arms, respectively, at 5.5-year follow-up.

[c] Controlled systolic blood pressure was defined as a systolic blood pressure of less than 140 mm Hg.

[d] Controlled blood pressure was defined as a systolic blood pressure of less than 140 mm Hg and a diastolic blood pressure of less than 90 mm Hg.

[e] Medication adherence refers to a perfect adherence with score of 0 based on the 4-item Morisky Green Levine Scale. Medication adherence was only measured among participants who were taking medicines (521 and 475 in intervention and control arms, respectively, and one participant is

© 2024 Tan J et al. *JAMA Network Open*.

missing data in intervention arm and one is missing data in control arm at 1-year follow-up; and 434 and 410 in intervention and control arms, respectively, at 5.5-year follow-up). Medication adherence outcomes were not adjusted for baseline outcome, since the set of participants taking medication at baseline was not the same set taking the medicine at follow-up.

[f] Disability was measured by the modified Rankin Scale, with scores ranging from 0 (no symptoms) to 5 (severe disability); people with a score of ≥3 were categorized into the "moderate to severe disability" group. The generalized estimating equations models for 1-year within-trial and 5.5-year post-baseline assessments did not converge, we used a marginal standardization approach by fitting a binomial model with a logit link to obtain risk differences.

[g] Stroke recurrence for 1-year within-trial assessment refers to any recurrent stroke in post-baseline 1 year, and for 5.5-year post-baseline assessment refers to any recurrent stroke in post-baseline 5.5 years. The generalized estimating equations model for 1-year within-trial assessment did not converge after we additionally adjusted month of interview, so we used a marginal standardization approach by fitting a binomial model with a logit link to obtain risk differences.

© 2024 Tan J et al. *JAMA Network Open*.

eTable 4. Additional Sensitivity Analyses

Outcomes, n (%)	Intervention (n=499)	Control (n=499)	Adjusted for post-trial follow-up duration[a]	Adjusted for baseline imbalance (n=998)[b]	Adjusted for imbalance related to 5.5-year post-baseline loss to follow-up and death (n=989)[c]	Adjusted for both types of imbalance (n=989)[d]	Adjusted for both types of imbalance and including outliers (987)[e]
					Mean difference (95% CI)		
**Blood pressure related outcomes[f]**							
Change in systolic BP (mm Hg), mean (SD)	-4.4 (23.3)	-2.2 (24.2)	-3.0 (-5.6 to -0.3)	-3.1 (-5.6 to -0.5)	-2.8 (-5.3 to -0.3)	-3.0 (-5.7 to -0.4)	-3.0 (-5.7 to -0.4)
Change in diastolic BP (mm Hg), mean (SD)	3.1 (11.4)	3.4 (11.1)	-1.0 (-2.3 to 0.3)	-1.2 (-2.5 to 0.1)	-1.1 (-2.4 to 0.2)	-1.1 (-2.4 to 0.2)	-1.1 (-2.4 to 0.2)
					Risk ratio (95% CI)		
Controlled systolic BP[g]	243 (49.0)	220 (44.3)	1.11 (0.99 to 1.24)	1.13 (1.01 to 1.26)	1.13 (1.02 to 1.26)	1.13 (1.02 to 1.25)	1.13 (1.02 to 1.26)
Controlled BP[h]	220 (44.4)	204 (41.4)	1.06 (0.94 to 1.19)	1.07 (0.97 to 1.19)	1.09 (0.98 to 1.22)	1.08 (0.97 to 1.20)	1.08 (0.97 to 1.20)
**Medication use and adherence**							
Taking antihypertensive medications	434 (87.0)	410 (82.2)	1.03 (0.99 to 1.08)	1.03 (0.99 to 1.08)	1.03 (0.98 to 1.08)	1.03 (0.98 to 1.08)	-[i]
Adherence to antihypertensive medications[j]	290 (66.8)	249 (60.7)	1.10 (0.99 to 1.22)	1.11 (1.00 to 1.23)	1.11 (1.00 to 1.22)	1.10 (1.00 to 1.22)	-
**Disability and stroke recurrence**							

© 2024 Tan J et al. *JAMA Network Open*.

Moderate to severe disability[k]	165 (33.1)	165 (33.1)	0.98 (0.82 to 1.17)	0.94 (0.79 to 1.11)	0.88 (0.74 to 1.04)	0.89 (0.75 to 1.05)	-
Stroke recurrence[l]	101 (20.7)	128 (26.8)	0.76 (0.60 to 0.96)	0.78 (0.61 to 0.99)	0.77 (0.61 to 0.98)	0.77 (0.61 to 0.98)	-

Abbreviations: CI, confidence interval; SD, standard deviation; BP, blood pressure.

[a] Adjusted for baseline outcome, township, sex, age, month of interview, and post-trial follow-up duration; removing outliers in systolic and diastolic blood pressure based on a priori decision to remove those that are more than 2 interquartile range above the third quartile or below the first quartile.

[b] Adjusted for baseline outcome, township, sex, age, month of interview, and variables noted to be differential by treatment arm at baseline (based on $p<0.05$; baseline diastolic blood pressure, having hypertension, having none of the assets asked about, taking anti-hypertensive medications); removing outliers in systolic and diastolic blood pressure.

[c] Adjusted for baseline outcome, township, sex, age, month of interview, and variables noted to be differential by loss to follow-up and death of 5.5-year post-baseline assessment (based on $p<0.05$; baseline health-related quality of life in utility, stroke duration since diagnosis, Body Mass Index, type of phone owned, whether sharing the phone with family members, smoking status, whether achieve health enhancing physical activity, stroke recurrence since diagnosis, disability, depression status, completion time of timed up and go test); removing outliers in systolic and diastolic blood pressure.

[d] Fully adjusted model. Adjusted for baseline outcome, township, sex, age, month of interview, and variables noted to be differential by treatment arm at baseline and loss to follow-up and death of 5.5-year post-baseline assessment; removing outliers in systolic and diastolic blood pressure.

[e] Fully adjusted model but included the outliers. Adjusted for baseline outcome, township, sex, age, month of interview, variables noted to be differential by treatment arm at baseline and loss to follow-up and death of 5.5-year post-baseline assessment.

[f] The means and standard deviations of changes in systolic and diastolic blood pressure by arms are data summary statistics, not model-estimated, and are based on removing outliers; and the numbers and proportions of controlled systolic blood pressure and controlled blood pressure also removed outliers. Systolic blood pressure outliers were removed resulting in 5 and 4 removed from intervention and control arms, respectively, at 1-year follow-up; and 3 and 2 removed from intervention and control arms, respectively, at 5.5-year follow-up. Diastolic blood pressure outliers were removed resulting in 3 and 3 removed from intervention and control arms, respectively, at 1-year follow-up; and 2 and 6 removed from intervention and control arms, respectively, at 5.5-year follow-up.

[g] Controlled systolic blood pressure was defined as a systolic blood pressure of less than 140 mm Hg.

© 2024 Tan J et al. *JAMA Network Open*.

[h] Controlled blood pressure was defined as a systolic blood pressure of less than 140 mm Hg and a diastolic blood pressure of less than 90 mm Hg.

[i] There was no removal of outliers for these variables hence no results here.

[j] Medication adherence refers to a perfect adherence with score of 0 based on the 4-item Morisky Green Levine Scale. Medication adherence was only measured among participants who were taking medicines (434 and 410 in intervention and control arms, respectively). Medication adherence outcomes were not adjusted for baseline outcome, since the set of participants taking medication at baseline was not the same set taking the medicine at follow-up.

[k] Disability was measured by the modified Rankin Scale, with scores ranging from 0 (no symptoms) to 5 (severe disability); people with a score of ≥3 were categorized into the "moderate to severe disability" group.

[l] Stroke recurrence refers to any recurrent stroke in post-baseline 5.5 years.

© 2024 Tan J et al. *JAMA Network Open*.

eTable 5. Sensitivity Analyses of Within-Trial and Long-Term Outcomes Among 969 Participants Who Completed All 3 Assessments[a]

	1-year within-trial assessment			5.5-year post-baseline assessment		
**Outcomes, n (%)**	**Intervention (n=489)**	**Control (n=480)**	**Mean difference (95% CI)**	**Intervention (n=489)**	**Control (n=480)**	**Mean difference (95% CI)**
**Blood pressure related outcomes[b]**						
Change in systolic BP (mm Hg), mean (SD)	-7.2 (18.3)	-5.3 (18.9)	-2.2 (-4.5 to 0.0)	-4.4 (23.3)	-2.3 (24.5)	-2.8 (-5.5 to -0.2)
Change in diastolic BP (mm Hg), mean (SD)	-4.1 (9.6)	-2.4 (9.6)	-2.4 (-3.4 to -1.3)	3.2 (11.3)	3.4 (11.1)	-1.1 (-2.4 to 0.2)
			**Risk ratio (95% CI)**			**Risk ratio (95% CI)**
Controlled systolic BP[c]	268 (55.1)	231 (48.5)	1.15 (1.03 to 1.28)	240 (49.4)	210 (43.9)	1.15 (1.01 to 1.30)
Controlled BP[d]	265 (54.8)	219 (46.2)	1.20 (1.06 to 1.34)	217 (44.7)	194 (40.9)	1.11 (0.98 to 1.25)
**Medication use and adherence**						
Taking antihypertensive medications	416 (85.1)	378 (78.8)	1.04 (0.99 to 1.09)	426 (87.1)	395 (82.3)	1.02 (0.98 to 1.07)
Adherence to antihypertensive medications[e]	309 (74.5)	243 (64.5)	1.14 (1.02 to 1.27)	285 (66.9)	238 (60.3)	1.12 (1.00 to 1.24)
**Disability and stroke recurrence**						
Moderate to severe disability[f]	82 (16.8)	127 (26.5)	0.41 (0.29 to 0.59)	163 (33.3)	160 (33.3)	0.93 (0.79 to 1.11)
Stroke recurrence[g]	18 (3.7)	38 (8.0)	0.41 (0.23 to 0.74)	101 (20.7)	128 (26.8)	0.77 (0.61 to 0.99)

Abbreviations: CI, confidence interval; SD, standard deviation; BP, blood pressure.

[a] Only included participants who completed the baseline, 12-month post-baseline, and 5.5-year post-baseline assessments. Adjusted for baseline outcome, township, sex, age, and month of interview, and removing outliers in systolic and diastolic blood pressure based on an *a priori* decision to remove those that are more than 2 interquartile range above the third quartile or below the first quartile.

[b] The means and standard deviations of changes in systolic and diastolic blood pressure by arms are data summary statistics, not model-estimated, and are based on removing outliers; and the numbers and proportions of controlled systolic blood pressure and controlled blood pressure also removed outliers. Systolic blood pressure outliers were removed resulting in 5 and 4 removed from intervention and control arms, respectively, at 1-year follow-up; and 3 and 2 removed from intervention and control arms, respectively, at 5.5-year follow-up. Diastolic blood pressure outliers were removed resulting in 3 and 3 removed from intervention and control arms, respectively, at 1-year follow-up; and 2 and 6 removed from intervention and

© 2024 Tan J et al. *JAMA Network Open*.

control arms, respectively, at 5.5-year follow-up.

[c] Controlled systolic blood pressure was defined as a systolic blood pressure of less than 140 mm Hg.

[d] Controlled blood pressure was defined as a systolic blood pressure of less than 140 mm Hg and a diastolic blood pressure of less than 90 mm Hg.

[e] Medication adherence refers to a perfect adherence with score of 0 based on the 4-item Morisky Green Levine Scale. Medication adherence was only measured among participants who were taking medicines. Medication adherence outcomes were not adjusted for baseline outcome, since the set of participants taking medication at baseline was not the same set taking the medicine at follow-up.

[f] Disability was measured by the modified Rankin Scale, with scores ranging from 0 (no symptoms) to 5 (severe disability); people with a score of ≥3 were categorized into the "moderate to severe disability" group. The generalized estimating equations model for 1-year within-trial assessment did not converge, we used a marginal standardization approach by fitting a binomial model with a logit link to obtain risk ratio.

[g] Stroke recurrence for 1-year within-trial assessment refers to any recurrent stroke in post-baseline 1 year, and for 5.5-year post-baseline assessment refers to any recurrent stroke in post-baseline 5.5 years. The generalized estimating equations model for 1-year within-trial assessment did not converge, we used a marginal standardization approach by fitting a binomial model with a logit link to obtain risk ratio.

© 2024 Tan J et al. *JAMA Network Open*.

eTable 6. Sensitivity Analysis of Long-Term Outcomes by Excluding 19 Participants Who Completed the 5.5-Year Post-Baseline Assessment in August 2023[a]

Outcomes, n (%)	Intervention (n=495)	Control (n=484)	Mean Difference (95% CI)
**Blood pressure related outcomes[b]**			
Change in systolic BP (mm Hg), mean (SD)	-4.5 (23.4)	-2.3 (23.7)	-3.0 (-5.6 to -0.4)
Change in diastolic BP (mm Hg), mean (SD)	3.0 (11.4)	3.4 (10.9)	-1.3 (-2.5 to 0.0)
			**Risk ratio (95% CI)**
Controlled systolic BP[c]	242 (49.2)	214 (44.4)	1.14 (1.01 to 1.28)
Controlled BP[d]	219 (44.6)	198 (41.3)	1.10 (0.98 to 1.23)
**Medication use and adherence**			
Taking antihypertensive medications	432 (87.3)	401 (82.9)	1.03 (0.99 to 1.08)
Adherence to antihypertensive medications[e]	289 (66.9)	242 (60.4)	1.11 (1.00 to 1.24)
**Disability and stroke recurrence**			
Moderate to severe disability[f]	164 (33.1)	162 (33.5)	0.93 (0.78 to 1.10)
Stroke recurrence[g]	101 (20.9)	122 (26.4)	0.79 (0.61 to 1.01)

Abbreviations: CI, confidence interval; SD, standard deviation; BP, blood pressure.

[a] Excluding 19 participants (four and 15 in intervention and control arms, respectively) who were interviewed in August 2023. Adjusted for baseline outcome, township, sex, age, and month of interview, and removing outliers in systolic and diastolic blood pressure based on a priori decision to remove those that are more than 2 interquartile range above the third quartile or below the first quartile.

[b] The mean and standard deviation of change in systolic and diastolic blood pressure by arms are data summary statistics, not model estimated, and are based on removing outliers; and the numbers and proportions of controlled systolic blood pressure and controlled blood pressure also removed outliers. Systolic blood pressure outliers were removed resulting in 3 and 2 removed from intervention and control arms, respectively, at 5.5-year follow-up. Diastolic blood pressure outliers were removed resulting in 2 and 6 removed from intervention and control arms, respectively, at 5.5-year follow-up.

[c] Controlled systolic blood pressure was defined as a systolic blood pressure of less than 140 mm Hg.

[d] Controlled blood pressure was defined as a systolic blood pressure of less than 140 mm Hg and a diastolic blood pressure of less than 90 mm Hg.

[e] Medication adherence refers to a perfect adherence with score of 0 based on the 4-item Morisky Green Levine Scale. Medication adherence was only measured among participants who were taking medicines. Medication adherence outcomes were not adjusted for baseline outcome, since the set of participants taking a given medication at baseline was not the same set taking the medicines at follow-up.

[f] Disability was measured by the modified Rankin Scale, with scores ranging from 0 (no symptoms) to 5 (severe disability); people with a score of ≥3 were categorized into the "moderate to severe disability" group.

[g] Stroke recurrence refers to any recurrent stroke in post-baseline 5.5 years.

© 2024 Tan J et al. *JAMA Network Open*.

eFigure 1. Timeline and Follow-Up Duration of the SINEMA Main Trial and Post-Trial

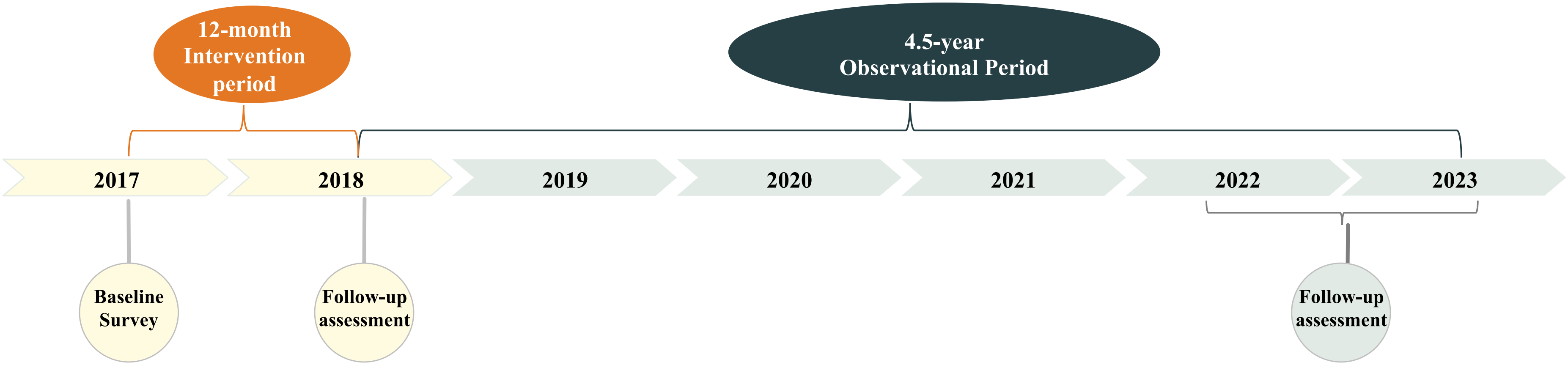

Month of data collection	No. (%) of participants		
	Intervention	Control	Total
**Baseline survey**			
June, 2017	224 (35.2%)	266 (40.2%)	490(37.7%)
July, 2017	413 (64.8%)	396 (59.8%)	809 (62.3%)
**Follow-up assessment**			
July, 2018	611 (100.0%)	608 (98.9%%)	1219 (99.4%)
August, 2018	0 (0.0%)	7 (1.1%)	7 (0.6%)
**Mean follow-up duration in months (SD)**	12.5 (0.2)	12.5 (0.2)	12.5 (0.2)

Month of data collection	No. (%) of participants		
	Intervention	Control	Total
**Post-trial assessment**			
October, 2022	341 (68.3%)	299 (59.9%%)	640 (64.1%)
May, 2023	154 (30.9%)	185 (37.1%)	339 (34.0%)
August, 2023	4 (0.8%)	15 (3.0%)	19 (1.9%)
**Mean follow-up duration in months (SD)**	66.2 (3.5)	67.0 (3.9)	66.6 (3.7)

© 2024 Tan J et al. *JAMA Network Open*.

eFigure 2. Mean Adjusted Differences in Systolic Blood Pressure Change From Baseline to 5.5-Year Follow-Up by Baseline Subgroup

Characteristics at baseline	Intervention		Control		Adjusted difference (95%CI)	P Value for Interaction
	No. of Participants	Mean (SD)	No. of Participants	Mean (SD)		
All participants	496	−4.4 (23.4)	497	−2.2 (24.2)	−2.8 (−5.3 to −0.3)	
Post−hoc subgroups						
Self−reported hypertension						0.09
Yes	354	−5.9 (23.9)	336	−1.8 (24.6)	−4.2 (−7.2 to −1.3)	
No	142	−0.8 (21.7)	161	−3.1 (23.5)	0.3 (−4.2 to 4.8)	
Stroke type						0.73
Ischemic stroke	433	−4.6 (23.5)	423	−1.8 (24.2)	−2.6 (−5.3 to 0.1)	
Hemorrhage stroke	61	−2.1 (22.1)	73	−4.3 (24.1)	−3.8 (−10.6 to 2.9)	
Duration since the first stroke event, years						0.98
≤5	277	−3.5 (23.5)	264	−1.9 (23.2)	−2.8 (−6.1 to 0.6)	
>5	219	−5.5 (23.2)	233	−2.6 (25.4)	−2.7 (−6.4 to 1.0)	
Systolic blood pressure, mmHg						0.41
<140	209	8.5 (18.4)	212	10.3 (19.3)	−1.6 (−5.3 to 2.0)	
≥140	287	−13.8 (22.1)	285	−11.5 (23.4)	−3.7 (−6.8 to −0.5)	
Moderate to severe disability						0.92
Yes	134	−5.5 (25.0)	113	−3.0 (25.5)	−3.0 (−8.0 to 2.0)	
No	361	−4.0 (22.7)	384	−2.0 (23.9)	−2.7 (−5.7 to 0.2)	
Recurrence of stroke since diagnosis						0.63
Yes	128	−3.8 (22.2)	141	−0.9 (24.1)	−1.8 (−6.4 to 2.8)	
No	368	−4.6 (23.8)	356	−2.8 (24.3)	−3.1 (−5.9 to −0.3)	

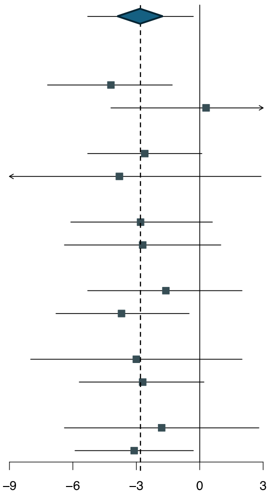

The between-arm mean differences (intervention − control) were estimated with a mixed-effects linear regression model, removing systolic blood pressure outliers based on an *a priori* decision to remove those that are more than 2 interquartile range above the third quartile or below the first quartile. The mixed effects model includes a random intercept for cluster (village) and fixed effects for baseline systolic blood pressure, township, sex, age, and month of interview, and interactions between intervention effect and subgroups. The mixed effects model did not converge for level of systolic blood pressure and recurrence of stroke since diagnosis subgroups, we employed the generalized estimating equations method with Gaussian model and an identity link. Analyses were performed for the 998 participants with 5.5-year follow-up (499 and 499 in intervention and control arms, respectively). Systolic blood pressure outliers were removed resulting in 3 and 2 removed from intervention and control arms, respectively. The mean and standard deviation of change in systolic blood pressure by arms and subgroups are data summary statistics, not model estimated, and removed outliers. The solid line represents no effect, and the dashed line represents the overall estimated effect.

© 2024 Tan J et al. *JAMA Network Open*.
