# Supplementary material for: Primary Care–Based Digital Health–Enabled Stroke Management Intervention: Long-Term Follow-Up of a Cluster Randomized Clinical Trial
Source: JAMA Netw Open. 2024 Dec 13;7(12):e2449561. doi: 10.1001/jamanetworkopen.2024.49561 (PMC11645652; doi:10.1001/jamanetworkopen.2024.49561)
Supplement: Supplement 3. — Data Sharing Statement [file jamanetwopen-e2449561-s003.pdf]

Data Sharing Statement

Tan. Primary Care–Based Digital Health–Enabled Stroke Management Intervention. *JAMA Netw Open*. Published December 10, 2024. doi:10.1001/jamanetworkopen.2024.49561

Data
Additional Information: Original trial: ClinicalTrials.gov NCT03185858. Follow-up study: ClinicalTrials.gov NCT05792618.
Data available: No

Additional Information
Explanation for why data not available: No patient-level data collected in this trial can be made available externally owing to internal regulations, patient consent, and data regulations. Yet, researchers interested in collaboration should contact the corresponding authors. Upon reasonable request, non-identifiable aggregated data could be shared pending approval from the steering committee and from the institutional review boards.
